# Broadening participation: 20-year outcomes from undergraduate science training programs

**DOI:** 10.1126/sciadv.aeh0739

**Published:** 2026-06-17

**Authors:** Anna Woodcock, Samantha D. Aguilar, Paul R. Hernandez, Mary E. Peterson, P. Wesley Schultz

**Affiliations:** ^1^ Division of Behavioral and Organizational Sciences, Claremont Graduate University, Claremont, CA, USA.; ^2^ Department of Educational Psychology, Texas A&M University, College Station, TX, USA.

## Abstract

Science thrives on diverse perspectives, yet women and scientists from underrepresented racial and ethnic groups remain underrepresented in doctoral-level biomedical research careers. Despite decades of investment in training initiatives, rigorous long-term evidence of their effectiveness is limited. We conducted a 20-year prospective, matched-control longitudinal study to test whether participation in two National Institutes of Health undergraduate programs, the Research Initiative for Scientific Enhancement (RISE) and Minority Access to Research Careers (MARC), increases doctoral (Ph.D.) completion. Using propensity score matching on 11 baseline characteristics, we followed 743 students from 2005 to 2025. Participants were more than twice as likely as matched peers to earn a Ph.D., with effects remaining robust after adjusting for prior academic achievement and career intentions. These findings demonstrate that sustained undergraduate training programs can durably expand participation in the biomedical research workforce.

## Teaser

Twenty-year study shows NIH training programs double Ph.D. completion among underrepresented students.

## INTRODUCTION

The biomedical research enterprise depends on a strong pool of students and trainees committed to research careers. Yet, despite sustained initiatives, women, African American, Hispanic/Latino, and Native American scientists remain markedly underrepresented in doctoral-level research positions (*1*). A larger and more diverse scientific workforce is essential for US security, economic competitiveness, and resilience to threats such as emerging infections and climate-driven disasters (*2*, *3*). Innovative strategies for recruitment and retention can expand participation, enabling the US biomedical research enterprise to realize its full potential.

For more than 50 years, US academic institutions have implemented intervention and training programs to broaden participation in science (*4*–*7*). Yet, during recent cuts to National Institutes of Health (NIH) and National Science Foundation (NSF) budgets, such programs were disproportionately reduced (*8*). Hundreds of minority training initiatives operated annually across campuses, but their efficacy remains uncertain and increasingly scrutinized (*9*). Most evaluations rely on data from a single program or program sites (*10*–*13*), counts of students served, program activities, or anecdotal accounts of presumed impact (*14*, *15*). Large-scale, systematic assessments using rigorous behavioral research methods and appropriate controls are notably absent, leaving little reliable evidence of outcomes. What is missing is an empirical foundation of theory-driven evaluation that can inform program design and funding decisions. Although studies suggest benefits of individual elements—such as mentoring, research experience, and financial support (*11*, *16*, *17*)—few prospective longitudinal studies exist to test their long-term effects in comparison to matched controls.

### Purpose of the study

The purpose of this study was to test the effectiveness of large-scale national undergraduate science training programs on subsequent doctoral degree completion. We report evidence from a 20-year prospective, matched-control national longitudinal study of training programs aimed at encouraging underrepresented minority undergraduates to pursue a research career in the biomedical sciences: The NIH’s Research Initiative for Science Enhancement (RISE) and Minority Access to Research Careers (MARC) mechanisms. Founded in 1972, RISE programs provide research support for faculty and students at minority-serving educational institutions. In 2005, when this study began, RISE programs typically received around $600,000 per year to support ~25 undergraduate and ~5 master’s-level graduate students, with the goal of increasing the number of doctoral-level minority biomedical researchers. MARC programs focused on preparing undergraduate honors students from underrepresented groups in their last 2 years of college for high-caliber scientific doctoral training.

Each grant-receiving campus has flexibility in structuring its programs and typically includes a combination of the following: faculty mentoring of students, on-campus research, graduate school preparation support, summer research internships, funding to attend and present at professional conferences, and direct financial support. The common feature of RISE, MARC, and many similar programs is their focus on providing mentorship and undergraduate research experience with a faculty member. Because of their longstanding history of funding, nationwide coverage, and breadth of program elements, the RISE and MARC programs provide an excellent context for our research. In this preregistered study (OSF: https://osf.io/5w7cy/), we test the hypothesis that participating in a RISE or MARC program as an undergraduate will significantly increase the likelihood of earning a doctoral degree. Data and output are available (https://doi.org/10.5061/dryad.dz08kpsc4).

## RESULTS

### Baseline group equivalence

One of the challenges to testing the effectiveness of a training program is identifying an appropriate comparison group. Because students are competitively selected into these training programs, it is likely that they were already on a path toward academic and career success. To address this methodological challenge, we used a prospective propensity score matching procedure to identify a matched sample of equally prepared and motivated students and then followed the full panel for more than 20 years. This is a key feature of our study and one that adds strength to our causal conclusions about the impact of program participation on academic and career outcomes.

We recruited students from universities with RISE/MARC and a pool of matched universities without such programs. We created a propensity score matched sample between undergraduates who were members of RISE or MARC programs (treatment group) and those who were not members of a RISE or MARC program (control group) (*n* = 743; see Materials and Methods for details). We matched the groups on 11 baseline variables: *sex*, *age*, *race*/*ethnicity*, *major*, *university*, *grade point average* (*GPA*), *educational progress*, *intention to become a research scientist*, *first language spoken*, *transfer status*, and *first-generation college status*. Preliminary analyses showed that at baseline, MARC students tended to be older, have higher *GPAs*, and had stronger *intentions to become a scientist* than did RISE students. These effects were expected, given the more selective nature of the MARC program; however, the matched control groups for each program did not differ significantly from their respective treatment group, indicating the effectiveness of the propensity score matching procedure. See Table 1.

**Table 1.** Baseline measures as a function of training program (*N* = 743). For categorical variables: Fr, frequency (%) variables: *P* values are from the Pearson chi-square test of independence. For continuous variables: mean (SD) variables: *P* values are from the analysis of variance (ANOVA) *F* test. Means sharing a common superscript letter (a, b, or ab) do not differ significantly (Tukey post hoc, *P* < 0.05). Asterisk (*) denotes statistical significance.

		RISE		MARC		
Measure	Group	Treatment Fr/M (%/SD)	Control Fr/M (%/SD)	Treatment Fr/M (%/SD)	Control Fr/M (%/SD)	Test
	Overall	329 (44.3%)	279 (37.6%)	68 (9.2%)	67 (9.0%)	
*Age*		20.99 (3.11)^a^	21.12 (2.80)^a^	22.72 (3.89)^b^	22.87 (4.75)^b^	<0.001*
*Sex*	Female	230 (70.2%)	191 (68.5%)	49 (72.1%)	48 (71.6%)	0.91
	Male	98 (29.8%)	88 (31.5%)	19 (27.9%)	19 (28.4%)	
*Race*/*ethnicity*	African American/Black	171 (52.0%)	148 (53.0%)	33 (48.5%)	32 (47.8%)	0.46
	Asian	15 (4.6%)	13 (4.7%)	2 (2.9%)	2 (3.0%)	
	Hawaiian/Pacific Islander	2 (0.6%)	3 (1.1%)	1 (1.5%)	3 (4.5%)	
	Hispanic/Latino/Latina	121 (36.8%)	100 (35.8%)	26 (38.2%)	27 (40.3%)	
	Native American/Alaskan Native	7 (2.1%)	2 (0.7%)	3 (4.4%)	-	
	White	13 (4.0%)	13 (4.7%)	3 (4.4%)	3 (4.5%)	
*English first language*	No	72 (22.0%)	69 (24.7%)	16 (23.5%)	14 (20.9%)	0.84
	Yes	256 (78.0%)	210 (75.3%)	52 (76.5%)	53 (79.1%)	
*First-generation college student*	No	256 (78.0%)	214 (76.7%)	54 (79.4%)	60 (89.6%)	0.14
	Yes	72 (22.0%)	65 (23.3%)	14 (20.6%)	7 (10.4%)	
*Transfer student*	No	282 (86%)	245 (87.8%)	48 (70.6%)	48 (71.6%)	0.63
	Yes	46 (14%)	34 (12.2%)	20 (29.4%)	19 (28.4%)	
*GPA*		3.32 (0.40)^a^	3.25 (0.39)^a^	3.57 (0.29)^b^	3.39 (0.42)^ab^	<0.001*
*Science research career intention*		8.65 (1.82)^a^	8.33 (1.77)^a^	9.27 (1.06)^b^	8.60 (1.57)^b^	<0.001*
*Major field of study*	Behavioral Sciences	25 (7.6%)	19 (6.8%)	9 (13.2%)	13 (19.4%)	
	Biological Sciences	201 (61.3%)	186 (66.7%)	33 (48.5%)	36 (53.7%)	0.07
	Engineering	9 (2.7%)	4 (1.4%)	3 (4.4%)	1 (1.5%)	
	Mathematics or Computer Science	6 (1.8%)	9 (3.2%)	2 (2.9%)	3 (4.5%)	
	Natural Sciences	82 (25.0%)	55 (19.7%)	18 (26.5%)	13 (19.4%)	
	Social Sciences	4 (1.2%)	6 (2.2%)	3 (4.4%)	1 (1.5%)	
	Other	1 (0.3%)	-	-	-	

### Nested data structure

To evaluate potential clustering in Ph.D. completion by institution, we first estimated a multilevel logistic regression model with a random intercept for college. The estimated variance of the random intercept was 0.0873, yielding an intraclass correlation coefficient (ICC) of approximately 2.6% [calculated as 0.0873/(0.0873 + π^2^/3)], indicating minimal clustering by institution. Given the low ICC, which falls below conventional thresholds for requiring multilevel modeling [e.g., 5 to 10%; (*18*)], we proceeded with single-level logistic regression models to test our hypotheses.

### Effects of the interventions

**Fig. 1.**
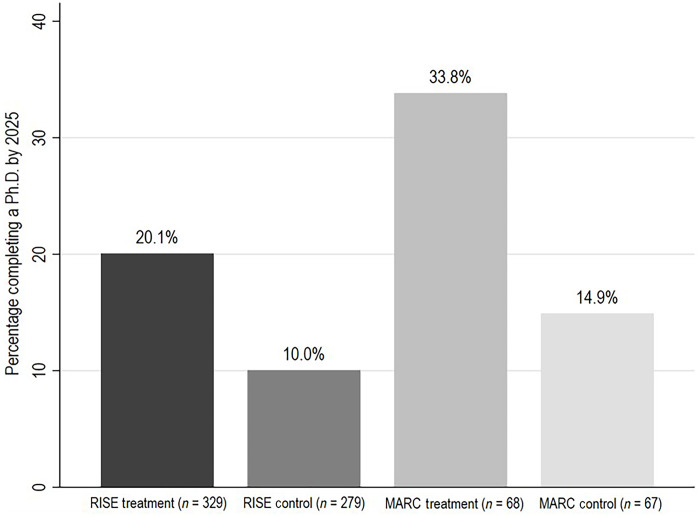
PhD attainment by RISE and MARC training program participation.

**Fig. 2.**
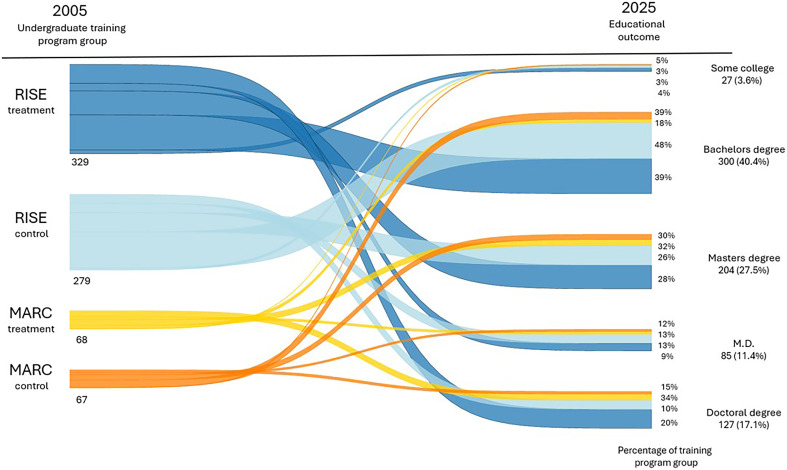
Educational attainment of RISE/MARC treatment and control group participants.

There are four categories of input: RISE treatment group (*n* = 329), RISE control group (*n* = 279), MARC treatment group (*n* = 68), and MARC control group (*n* = 67). Notably, treatment group participants were more than twice as likely to complete a Ph.D. as the control group. Approximately 20% of the RISE treatment group and 34% of the MARC treatment group students completed a Ph.D., compared with 10 and 15% of their respective comparison group students (Fig. 1). Figure 2 visualizes the movement of treatment and control group students in 2005 toward their ultimate academic outcomes by 2025.

To rigorously evaluate the relationship between program participation and Ph.D. attainment, we conducted logistic regression analyses. Initial unadjusted models comparing each intervention group to matched controls indicated that RISE treatment participants were over twice as likely to earn a Ph.D. [odds ratio (*OR*) = 2.25] and MARC treatment participants nearly three times as likely (*OR* = 2.91) than their matched counterparts. Collapsing across programs, treatment group membership was associated with significantly increased odds of Ph.D. completion (*OR* = 2.34, *P* < 0.001).

Adjusted models confirmed that both science career intention (*OR* = 1.25, *P* < 0.05) and GPA (*OR* = 2.36, *P* < 0.001) were significant predictors of Ph.D. attainment. Critically, treatment effects remained strong across models, with participants more than twice as likely to complete a Ph.D. compared to controls—even when both factors were jointly controlled (*OR* = 2.06, *P* < 0.001), reflecting an approximately 67% likelihood of completion: reinforcing the distinct and sustained impact of the programs.

## DISCUSSION

Our findings provide rare, prospective evidence that large-scale, targeted undergraduate training programs can move the needle on diversifying the US biomedical research workforce. Following undergraduates for 20 years across two national initiatives, RISE and MARC, we observed substantially higher Ph.D. completion rates among participants than matched controls. These differences remained robust after adjusting for science career intention and GPA, suggesting that the observed effects are unlikely to be explained by preexisting academic preparation or initial aspirations. These gains are consistent with mechanisms emphasized in prior research that are common to both programs: sustained mentorship, authentic research engagement, structured graduate preparation, professional socialization, and direct financial support.

Our baseline matching strategy relied on 11 observed variables, and we cannot exclude the possibility that unmeasured factors influenced both program participation and long-term outcomes. Although the MARC and RISE programs recruit highly motivated students, this selection alone does not ensure the pursuit or completion of a doctoral degree. Many students express strong intentions to pursue a scientific career during college but do not ultimately complete a Ph.D. The substantially higher Ph.D. completion rates among MARC and RISE students (relative to similar peers) likely reflect both selective recruitment and programmatic supports that prepare students to persist through the lengthy doctoral training process.

As with any long-term longitudinal study, attrition occurred between initial recruitment and the final analytic sample. Although loss is unavoidable over 20 years, missing data analyses (see Materials and Methods) suggest that differential attrition is unlikely to account for the observed differences in doctoral attainment. Nevertheless, future studies would benefit from expanded use of administrative datasets to further minimize missing outcome information.

With this in mind, we believe that our study provides the strongest evidence to date regarding the long-term impact of RISE and MARC on diversification of the biomedical research community. The prospective design, national scope, extended follow-up period, and consistency of effects across two initiatives collectively strengthen confidence in the findings.

For these findings to inform workforce diversification at scale, several steps are necessary. First, stable, multiyear funding is essential as the 20-year horizon of observed effects underscores that diversification is a long-term investment unlikely to succeed under short funding cycles. Second, rigorous evaluation should be embedded within funded programs or at the agency level, including standardized baseline measures, appropriate comparison strategies, and administrative data linkages to verify long-term outcomes. Third, scaling must prioritize institutional capacity-building to ensure that mentorship quality, research engagement, and graduate preparation remain strong as programs expand. Last, agencies and universities should align accountability metrics with long-term doctoral and research career outcomes rather than short-term participation counts.

Together, these implementation priorities translate the present evidence into actionable policy guidance and clarify the conditions under which similar gains can be realized more broadly. The magnitude and durability of the observed gains in doctoral degree attainment argue for protecting and expanding evidence-based training. Targeted and sustained undergraduate interventions provide a practical, empirically supported route to a more inclusive and thus more innovative and resilient US biomedical research enterprise.

## MATERIALS AND METHODS

### Experimental design

**Fig. 3.**
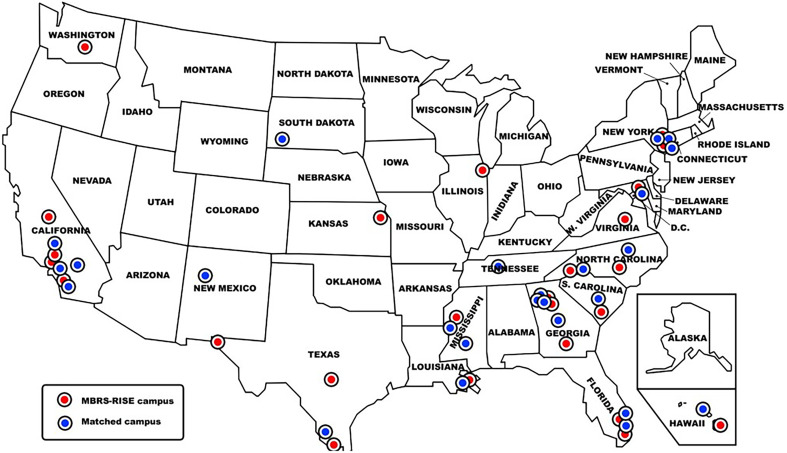
RISE/MARC treatment and control campuses.

This study uses a longitudinal quasi-experimental design. In 2005, we recruited a panel of 1420 predominantly Hispanic/Latino(a) and African American/Black students who either were or were not participating in a RISE or MARC science training program. Students were recruited from 25 RISE and/or MARC campuses and 25 non-RISE and MARC campuses, matched on the basis of geographic location, minority serving institution status, and Carnegie classification across the US (see Fig. 3).

Because the focus of the present analysis is undergraduate participation in MARC/RISE, 184 graduate students identified at baseline were excluded, yielding an undergraduate analytic base of *n* = 1236. To preserve internal validity, exclusions were then applied sequentially to eliminate treatment misclassification and funding heterogeneity: A total of 203 students coded as “EXTRA” in administrative records (*n* = 1033), 127 treatment students funded outside MARC/RISE mechanisms (*n* = 906), and 131 matched comparison students supported by alternative funding sources (*n* = 775). The resulting sample comprises 775 undergraduates with unambiguous treatment or matched comparison status.

### Missingness and attrition

Of the 775 eligible undergraduates, 743 (95.9%) were retained in the analytic sample. Baseline differences between included (*n* = 743) and excluded (*n* = 32) participants were assessed using *t* tests and chi-square tests. No statistically significant differences emerged for age, GPA, research career intention, gender, first language, transfer status, first-generation status, race/ethnicity, or major field. A logistic regression model predicting analytic inclusion from these baseline covariates was not statistically significant (overall model *P* = 0.18), and no individual predictors reached significance. Together, these results indicate that attrition was not systematically associated with treatment assignment or observed baseline characteristics, supporting the assumption that missingness is consistent with missing at random (MAR).

### Degree verification and final analytic sample

Doctoral attainment was verified using 2025 data from the National Student Clearinghouse (NSC), with retrospective review of enrollment and degree records spanning 2005 to 2025. NSC administrative records were cross-checked with longitudinal self-reports collected across study waves to determine each participant’s highest degree attained.

Among the 775 eligible undergraduates, 743 students (95.9%) had verifiable highest degree outcomes. For 32 students (4.1%), degree completion status could not be reliably determined on the basis of available records; these individuals were excluded from analysis. The final analytic sample therefore consists of *n* = 743 participants.

The reduction from the original 1420 participants reflects restriction to (i) undergraduate students only, (ii) clearly classified MARC/RISE treatment or matched comparison participants, and (iii) individuals with verified highest-degree outcomes. These exclusions were implemented as methodological safeguards to ensure valid treatment classification, comparable analytic groups, and accurate measurement of doctoral attainment.

### Matched participants

A 1:1 propensity score matched sample was created between students who were members of RISE or MARC programs (treatment group) and those who were not members of a RISE or MARC program (control group). The groups were matched on the following criteria: *sex*, *age*, *race*/*ethnicity*, *major*, *university*, *GPA*, *educational progress*, *intention to become a research scientist*, *first language spoken*, *transfer status*, and *first-generation college status*. The analytic sample of 743 reported here represents 397 treatment group participants and 346 control group members for whom we have outcome data for in 2025. The final sample consisted of 70% female, 52% African American/Black, 37% Hispanic/Latino, 61% biological sciences and 23% natural sciences majors, 33% juniors and 39% seniors, with an average age of 21.36 years, GPA of 3.32, and intention to pursue a scientific research career of 8.58 on a 0 to 10 scale. See Table 1 for sample demographics by group.

### Data collection

Online survey data were collected twice per year during the spring and fall over 24 consecutive waves from 2005 to 2017 and 2 additional consecutive waves in 2024. Using the Tailored Panel Management approach (*19*), we garnered per-survey response rates of ≥70% through 2017. Survey data were augmented by verified degree award data from the NSC (https://studentclearinghouse.org/) data warehouse, matched to each participant. More than 92% of accredited US colleges and universities submit enrollment and degree award data to the NSC, providing the most comprehensive source of verified student enrollment and degree attainment data.

### Measures

#### Training program intervention

We assigned participants to one of four groups as follows: RISE (i), RISE control (ii), MARC (iii), or MARC control (iv), with control groups matched via propensity scores. We recoded these groups into a binary variable: Training program participants (treatment = 1) and matched nonparticipants (control = 0).

#### Ph.D. completion

Doctoral attainment was defined strictly as the completion of a Ph.D., consistent with NIH criteria for scientific research careers, which exclude clinical and professional doctorates (NIH, 2023). From 2005 to 2025, participants reported their highest degree attained at each time point. We verified and augmented self-report data with verified data from the NSC. Those reporting a Ph.D. by 2025 were coded as having completed a doctorate (Ph.D. = 1); all others were coded as not having done so (Ph.D. = 0).

#### Baseline measures

Baseline surveys collected *age*, *binary sex* (male/female), and *ethnicity* [e.g., African American/Black, Asian, Hispanic/Latino/Latina, Native American/Alaska Native, white (non-Hispanic)], *GPA*, along with *English as a Second Language status*, *transfer student status*, and *first-generation college status* (each coded 1 = Yes, 0 = No). Participants also indicated their *major field*, selecting from seven predefined science, technology, engineering, and mathematics (STEM) categories. Participants were asked a single question: “On a scale of 0 to10, to what extent do you intend to pursue a science-related research career?” (*M* = 8.58, *SD* = 1.74).

### Statistical analysis

All analyses were conducted using Stata v.18 (StataCorp LLC, 2023). Missing data were coded to distinguish types of nonresponse and treated as missing in all analyses. The analytic sample and subsample sizes are as follows: Total *N* = 743; RISE treatment (*n* = 329), RISE control (*n* = 279), MARC treatment (*n* = 68), and MARC control (*n* = 67).

Baseline equivalence between analytic and excluded participants was assessed using independent-samples *t* tests for continuous variables and Pearson chi-square tests for categorical variables. These analyses evaluated whether participants retained in the analytic sample differed from those excluded on baseline characteristics, such as *GPA*, *science career intention*, *demographic characteristics*, and *academic background*, which could indicate potential selection bias.

#### Program effects on Ph.D. completion

The primary outcome variable was Ph.D. completion by 2025 (1 = completed, 0 = not completed). Initial comparisons between treatment and matched control groups were conducted using Pearson chi-square tests of independence. These tests evaluated whether Ph.D. completion rates differed between program participants and their matched control groups.

Separate pairwise comparisons were conducted for each program:

1) RISE treatment versus RISE matched control

2) MARC treatment versus MARC matched control

ORs were calculated from 2 × 2 contingency tables to quantify the magnitude of association between program participation and doctoral completion.

#### Logistic regression models

Logistic regression models were estimated to examine the association between program participation and the probability of Ph.D. completion while allowing focal contrasts among program groups. Participants were categorized into four groups: RISE treatment, RISE control, MARC treatment, and MARC control. Program contrasts were implemented using orthogonal contrast coding to directly test the following comparisons:

1) RISE treatment versus RISE control

2) MARC treatment versus MARC control

3) Average of RISE groups versus average of MARC groups

Models were estimated using maximum likelihood estimation. Logistic regression was used because the outcome variable was binary, and the model estimates the association between program participation and baseline characteristics and the odds of completing a Ph.D. Results are reported as ORs with corresponding Wald statistics, two-tailed *P* values, and 95% confidence intervals. Wald statistics were used to evaluate whether individual regression coefficients differed significantly from zero.

Adjusted models (*N* = 729 due to missing covariate data) included baseline covariates measured at Wave 0, including *GPA*, *science career intention*, *gender*, *age*, *race*/*ethnicity*, *major field*, *English as first language*, *transfer status*, and *first-generation college status*. Exploratory models examined interactions with the academic field. Continuous predictors were mean-centered before analysis to improve interpretability and reduce potential multicollinearity. Model fit was evaluated using the Hosmer-Lemeshow goodness-of-fit test, which assesses the agreement between observed and predicted probabilities of the outcome.

#### Clustering by institution

Because participants were nested within undergraduate institutions, we assessed whether multilevel modeling was necessary by estimating a multilevel logistic regression model with a random intercept for institution. This model allowed the baseline probability of Ph.D. completion to vary across institutions. The estimated variance of the institutional random intercept was 0.0873. The ICC was calculated using the logistic-link approximation

$$\text{ICC}=\left(\partial^{2}\text{instituion}\right)/\left[\sigma^{2}\text{institution}+\left(\pi^{2}\right)/3\right]$$

where $\sigma^{2}\text{institution}$ = estimated variance between institutions (from the random intercept), and $\frac{\pi^{2}}{3}$ = assumed variance at the individual level in logistic regression.

The ICC was calculated to determine the proportion of variance attributable to institutional clustering. ICCs below 5 to 10% were considered insufficient to warrant multilevel modeling; therefore, subsequent analyses used single-level logistic regression models.
